# Culture-based bacterial evaluation of the appendix lumen and antibiotic susceptibility of acute appendicitis in Japan: A single-center retrospective analysis

**DOI:** 10.1097/MD.0000000000039037

**Published:** 2024-07-19

**Authors:** Hiroe Kitahara, Yonfan Park, Kai Seharada, Masaki Yoshimura, Akira Horiuchi, Yukihiko Karasawa

**Affiliations:** a Department of Surgery, Showa Inan General Hospital, Komagane, Japan; b Department of Gastrointestinal Surgery, Shinshu University School of Medicine, Nagano, Japan; c Department of Surgery, Shinshu Ueda Medical Center, Nagano, Japan; d Digestive Disease Center, Showa Inan General Hospital, Komagane, Japan.

**Keywords:** acute appendicitis, antibiotic, appendix lumen, bacteria culture, extended-spectrum β-lactamase

## Abstract

The question of whether to perform an appendectomy or conservative treatment for acute appendicitis can differ depending on the facility or surgeon, but antibiotic treatment is administered regardless of whether an appendectomy or conservative treatment is selected. We investigated the contemporary bacteriology for acute appendicitis and evaluated the antibiotic sensitivity of the bacteria that are currently associated with appendicitis. We retrospectively analyzed the bacterial culture results and antibiotic susceptibility of 141 patients who underwent appendicitis surgery, including the identification and antimicrobial susceptibility of the cultured bacteria within the excised appendices. Bacterial cultures were positive in 131 cases (92.9%). The most commonly isolated bacteria were *Escherichia coli* (90 isolates, 66.7%), followed by *Enterococcus species* (n = 19, 14.5%), *Pseudomonas aeruginosa* (n = 18, 13.7%), *Streptococcus species* (n = 15, 11.5%), and *Klebsiella species* (n = 8, 6.1%). Eight strains (8.8%) of *E coli* were extended-spectrum β-lactamase producers, and ten strains (11.1%) were fluoroquinolone-resistant. Tazobactam/piperacillin and meropenem inhibited the growth of 100% of the major identified bacteria. The patients with appendicoliths had a significantly higher bacterial culture rate. *Enterococcus species* were frequently isolated from the patients with complicated appendicitis. For the antibiotic treatment of appendicitis, it is essential to understand the patient’s microbiological profile and antibiotic susceptibilities. Research from Asian countries such as Japan can enhance our knowledge of regional antibiotic resistance patterns and inform effective treatment strategies.

## 1. Introduction

Acute appendicitis is a common disease that often requires emergency surgery. According to a systematic review with a meta-analysis, surgical resection continues to be the preferred treatment, although antibiotic therapy alone may be considered for specific patients.^[1]^ Antibiotics-only treatment has usually involved broad-spectrum antibiotics, including ertapenem and combinations of multiple antibiotics. Although this approach can effectively target the primary microorganisms that are responsible for acute appendicitis, there are concerns about the potential development of multidrug resistance; given the global tendency to overuse antibiotics, this is a worrisome issue.

The selection of appropriate antibiotics for acute appendicitis is often empirical, as obtaining a culture specimen is challenging unless the peritoneal cavity is accessed (except in cases of bacteremia). Common bacteria in appendicitis encompass *Escherichia coli*, *Bacteroides*, *Streptococci*, and *Pseudomonas*.^[2]^ In Japan, there are no specific guidelines for the use of antibiotics in the treatment of acute appendicitis. Notably, widely accepted global guidelines such as those from the Surgical Infection Society and the Infectious Disease Society of America (SIS-IDSA)^[3]^ and the World Society of Emergency Surgery (WSES)^[4]^ cannot be directly applied in Japan and other Asian countries due to the high prevalence of extended-spectrum β-lactamase (ESBL)-producing and fluoroquinolone-resistant *E coli* in this region. The cautious selection of empirical antibiotics is therefore essential.

We conducted the present study to delve into the microbiology of patients diagnosed with acute appendicitis who underwent treatment with surgery combined with antibiotic therapy. The primary aims of the study were to identify the bacteria commonly found in the appendix lumen during acute appendicitis and to evaluate the susceptibility of these bacteria to various antibiotics.

## 2. Materials and methods

We retrospectively reviewed the medical records of the patients who underwent appendectomy and were histopathologically diagnosed as having acute appendicitis at Showa Inan General Hospital (Komagane, Japan) between April 2014 and September 2022. Patients who underwent interval appendectomy and those without intraoperative cultures of the lumen of the excised appendix were excluded from the study. At our hospital, the diagnosis of appendicitis is comprehensively determined based on clinical findings, blood sampling data, and imaging (computed tomography or ultrasonography). All resected appendices in the present series were evaluated histopathologically. Laparoscopic appendectomy is typically performed for patients who are eligible for surgery at our hospital, whereas open laparotomy is performed in cases with extensive abscesses. We performed the patients’ surgeries by the single-incision plus one-port laparoscopic method, with an additional port added depending on intraoperative findings. Aspiration or swabbing of the lumen of the excised appendix was performed in each patient. The swabbing was performed using Seed-Swab γ-3 (Eiken Chemical Co., Ltd., Tokyo, Japan), a flocked swab with a modified liquid Amies microbiology transport medium. These specimens were sent for culture and sensitivity testing to identify bacterial growth and the assessment of antibiotic sensitivity.

Each culture was subjected to antimicrobial susceptibility testing in our hospital’s clinical laboratory. The bacteria were cultured on blood agar, chocolate agar, Brucella HK agar, and bromothymol blue lactate agar. The autoed MicroScan WalkAway 40plus (Beckman Coulter, Brea, CA) was used to identify pathogens and perform the antibiotic susceptibility tests. Based on the susceptibility reports, antibiotics considered to be effective were then identified. The antimicrobial susceptibility tests were performed according to the criteria of the Clinical and Laboratory Standards Institute.

The timing of the surgery depended on the decision of the physician in charge at the patient’s first visit or the surgeon in charge of the surgery. Emergency surgery was performed in cases in which antibiotic treatment was administered but the patient’s symptoms worsened after his or her admission.

The following parameters were evaluated: the patients’ preoperative characteristics, including age, sex, antibiotic administration status, and time from diagnosis to surgery; the surgical procedure, including open or laparoscopic appendectomy; the histological or clinical classification of appendicitis, including phlegmonous and gangrenous; and bacterial cultures with antibiotic susceptibility, including specimens of bacterial cultures obtained by aspirating or swabbing the lumen of the excised appendix specimen.

Statistical analyses were performed with JMP ver. 15.2.0 (SAS Institute, Cary, NC). Fisher exact test was used as appropriate for the statistical analysis. Statistical significance was considered as a *P* value < .05. The study was approved by the institutional review board of Showa Inan General Hospital (No. 2021-06). The requirement for patients’ written informed consent was waived because this was a retrospective study. This study was conducted in accord with the STROBE guidelines and with the latest version of the Declaration of Helsinki. All authors had access to the study data and reviewed and approved the final manuscript.

## 3. Results

During the study period, a total of 320 patients were admitted to our hospital with acute appendicitis. Among them, 209 patients underwent appendectomy, and appendiceal lumen culture testing was conducted in 141 patients (Table 1). The ages of the patients (82 [58%] men, 59 [42%] women) ranged from 3 to 94 years (mean 41 years). The result of a bacterial culture was positive in 131 cases (93%). In all patients, intravenous antibiotics were administered from the day of admission. The initial antibiotic was cephem. Of the cases in which cephem was selected, ceftriaxone was used in 5 patients, and cefmetazole (CMZ) was used in the other 136 patients. Twenty-four patients who underwent surgery within 4 hours of their visit were not given preoperative antibiotics. The remaining 117 patients received preoperative antibiotic administration.

**Table 1:** The patients’ characteristics.

Age, yr	41.0 (3–94)
Gender, n (%)	
Male	82 (58.2)
Female	59 (41.8)
Preoperative in-hospital stay, d	0 (0–8)
Histopathological findings	
Catarrhal appendicitis	6 (4.3)
Phlegmonous appendicitis	90 (63.8)
Gangrenous appendicitis	22 (15.6)
Perforated appendicitis	23 (16.3)
Appendicolith	74 (52.5)
Procedure	
Laparoscopic appendectomy	140 (99.3)
Open appendectomy	1 (0.7)
Operation time, min	65 (27–206)
Postoperative hospital stay, d	3 (1–49)
SSI	17 (12.1)
Superficial incisional SSI	13 (9.2)
Postoperative intra-abdominal infection	5 (3.5)
Bacterial culture-positive patients	131 (92.9)

The data are the median (range) or number (%). SSI = surgical site infection.

The preoperative in-hospital stay ranged from 0 to 8 days (mean 0 day), and 122 patients underwent the appendicitis surgery within 24 hours of admission. Antibiotic treatment for ≥24 hours before surgery was administered to 19 patients. During the in-hospital waiting period, 3 patients were deemed to have worsened or were deemed unable to wait based on their physical assessments, necessitating emergency surgery. A total of 140 patients (99%) underwent laparoscopic appendectomy, and the remaining patient (1%) underwent an open appendectomy. Based on their intraoperative and pathological findings, 45 patients were diagnosed with complicated appendicitis.

The histopathological findings were as follows: catarrhal appendicitis, n = 6 patients (4%); phlegmonous appendicitis, n = 90 (64%); gangrenous appendicitis, n = 22 (16%); and perforated appendicitis, n = 23 (16%). A surgical site infection (SSI) occurred in 17 patients. Five patients developed a postoperative intra-abdominal infection, and 13 patients had a superficial incisional SSI. One patient experienced both an intra-abdominal infection and a superficial incisional SSI. Two patients with a postoperative intra-abdominal infection required conservative treatment with antibiotics, and 3 others needed interventional procedures or reoperations (Table 1).

The most commonly isolated bacteria were *E coli* (90 isolates, 66.7%), followed by *Enterococcus species* (n = 19, 14.5%), *Pseudomonas aeruginosa* (n = 18, 13.7%), *Streptococcus species* (n = 15, 11.5%), and *Klebsiella species* (n = 8, 6.1%). More than 2 organisms were isolated in 75 patients (57.3%). Eight strains (8.8%) of *E coli* were ESBL producers (Table 2).

**Table 2:** Bacterial isolates from 131 patients.

Bacterium	n (%)
*Escherichia coli*	90 (66.7)
Non-ESBL	82 (62.6)
ESBL	8 (6.1)
*Enterococcus species*	19 (14.5)
*Pseudomonas aeruginosa*	18 (13.7)
*Streptococcus species*	15 (11.5)
*Klebsiella pneumonia*	8 (6.1)
*Citrobacter*	5 (3.8)
*Enterobacter species*	3 (2.3)
*Staphylococcus species*	3 (2.3)
*Bacillus species*	3 (2.3)
*Proteus species*	2 (1.5)
*Bacteroides species*	1 (0.7)
*Nonfermenter species*	1 (0.7)
*Serratia marcescens*	1 (0.7)
Other Gram-negative rod	14 (10.7)
Other Gram-positive cocci	9 (6.9)

ESBL = extended-spectrum β-lactamase.

The results of the antimicrobial susceptibility test of the 5 major identified bacteria are shown in Table 3. Among the most commonly identified bacteria, *E coli* (non-ESBL) exhibits notable susceptibility to a wide array of antibiotics. The following effectively inhibited the bacterial growth of *E coli* (non-ESBL): CMZ (82/82 bacteria; 100%), tazobactam/piperacillin (TAZ/PIPC) (82/82 bacteria; 100%), meropenem (MEPN) (82/82 bacteria; 100%), and amikacin (82/82 bacteria; 100%). Ten strains (11.1%) were levofloxacin-resistant.

**Table 3:** Antibiotic susceptibility of major identified bacteria.

		Susceptibility												
Bacteria	Cases, n	Ampicillin	Sulbactam/ampicillin	Tazobactam/piperacillin	Cefazolin	Cefotiam	Cefmetazole	Cefepime	Meropenem	Clindamycin	Amikacin	Gentamicin	Levofloxacin	Vancomycin
*Escherichia coli*	90													
(Non-ESBL)	82	60/22 (72.2)	64/4 (77.1)	82/0 (100)	82/0 (100)	82/0 (100)	82/0 (100)	82/0 (100)	82/0 (100)	–	82/0 (100)	77/5 (93.9)	71/10 (86.6)	–
(ESBL)	8	8/0 (100.0)	3/1 (37.5)	8/0 (100)	0/8 (0.0)	0/8 (0.0)	8/0 (100)	0/8 (0.0)	8/0 (100.0)	–	8/0 (100.0)	5/3 (62.5)	4/4 (50.0)	–
*Enterococcus*	19	12/7 (63.2)	–	–	–	–	–	–	6/1 (31.6)	–	–	-	18/0 (94.7)	18/0 (94.7)
*Pseudomonas*	18	10/18 (55.6)	–	18/0 (100)	–	–	–	18/0 (100)	18/0 (100)	–	17/0 (94.4)	13/3 (72.2)	17/0 (94.4)	–
*Streptococcus*	14	14/0 (100)	–	–	–	2/0 (14.3)	–	11/0 (78.6)	13/0 (92.6)	8/2 (57.1)	13/0 (92.6)	–	–	14/0 (100)
*Klebsiella*	8	1/6 (12.5)	7/0 (87.5%)	8/0 (100.0)	71 (87.5%)	8/0 (100.0)	8/0 (100.0)	8/0 (100.0)	8/0 (100.0)	–	8/0 (100.0)	8/0 (100.0)	8/0 (100.0)	–

The data are (%). R: natural resistance to antibiotics, –: data not evaluated. ESBL = extended-spectrum β-lactamase.

The ESBL-producing *E coli* displayed resistance to cefotiam and cefepime. Sulbactam/ampicillin showed limited efficacy for inhibiting the growth of ESBL-producing *E coli* (3/8 bacteria; 37.5%). On the other hand, CMZ (8/8 bacteria; 100%), TAZ/PIPC (8/8 bacteria; 100%), and MEPN (8/8 bacteria; 100%) effectively suppressed the bacterial growth of ESBL-producing *E coli*. *P aeruginosa* showed reduced susceptibility to amikacin (17/18 bacteria; 94.4%) and levofloxacin (17/18 bacteria; 94.4%) but demonstrated susceptibility to TAZ/PIPC (18/18 bacteria; 100%) and MEPN (18/18 bacteria; 100%). Based on the present results, the identified major bacteria were almost completely susceptible to MEPN and TAZ/PIPC. In terms of aminoglycoside, some bacteria were more susceptible to amikacin than gentamicin, including *E coli* (amikacin 100% vs gentamicin 83.3%) and *P aeruginosa* (amikacin 94.4% vs gentamicin 72.2%). Other than *E coli*, no bacteria showed any problematic antibiotic resistance (Table 3).

The bacterial culture was positive in 131 patients (92.9%). The bacterial culture rate decreased significantly in the group of patients who underwent antibiotic treatment for >24 hours before their surgery. Preoperative antibiotic administration, especially for durations exceeding 24 hours, appears to correlate with a decreased positivity rate in bacterial cultures. The group of patients with appendicoliths had a significantly higher bacterial culture rate. Neither complicated appendicitis nor SSI was associated with a positive culture (Table 4).

**Table 4:** Associations between patient characteristics and culture-positive rates.

Bacterial culture	Positive (%)	Negative (%)	*P* value
Antibiotic treatment before surgery	14 (77.8)	117 (95.1)	.024
Appendicolith	73 (98.7)	58 (86.6)	.006
Complicated appendicitis	45 (93.7)	86 (92.4)	1.000
SSI	14 (93.3)	117 (92.8)	1.000

SSI = surgical site infection.

When preoperative antibiotic treatment was administered for >24 hours, the detection rate of *E coli* (non-ESBL) decreased to 26%. *E coli* (ESBL) decreased, but the difference was not significant. However, *Enterococcus species*, *P aeruginosa, Streptococcus species*, and *K pneumoniae* did not show a decrease even with preoperative antibiotic treatment for >24 hours (Table 5).

**Table 5:** Detection rate of bacteria in the patients with or without antibiotic treatment for ≥24 hours before surgery.

Bacterium	Antibiotic treatment (%)	Absent (%)	*P* value
*Escherichia coli* (non-ESBL)	5 (26.3)	77 (63.1)	.004
*Escherichia coli* (ESBL)	3 (17.6)	5 (4.0)	.075
*Enterococcus species*	5 (11.7)	14 (2.1)	.138
*Pseudomonas aeruginosa*	1 (5.2)	17 (13.9)	.467
*Streptococcus species*	0	15 (12.3)	.222
*Klebsiella pneumoniae*	1 (5.2)	7 (5.7)	1.000

ESBL = extended-spectrum β-lactamase.

*Enterococcus species* were detected significantly more frequently in patients with complicated appendicitis (22.9%) compared to those with simple appendicitis (8.6%). There were no significant differences in the detection of other bacteria between the simple appendicitis and complicated appendicitis cases (Table 6).

**Table 6:** Microorganisms cultured from the simple appendicitis and complicated appendicitis patients.

Bacterium	Simple appendicitis	Complicated appendicitis	*P* value
*Escherichia coli* (non-ESBL)	54 (58.1)	28 (58.3)	.580
*Escherichia coli* (ESBL)	5 (5.3)	3 (6.2)	1.000
*Enterococcus species*	8 (8.6)	11 (22.9)	.035
*Pseudomonas aeruginosa*	11 (11.8)	7 (14.5)	1.000
*Streptococcus species*	10 (10.7)	5 (10.4)	1.000
*Klebsiella pneumonia*	8 (7.5)	7 (2.1)	.265

ESBL = extended-spectrum β-lactamase.

## 4. Discussion

We retrospectively analyzed the microbiological profiles and antibiotic susceptibilities of appendicitis in a series of 141 patients. *E coli* was the most common pathogen identified (68.7% of all isolates), similar to previous appendicitis findings.^[2,5,6]^ The ratio of ESBL-producing *E coli* was 8.8%, which is within the reported range 5.3% to 16.8%.^[7–9]^ *Enterococcus species and Streptococcus species* were the most frequently isolated Gram-positive organisms.^[5]^ *P aeruginosa* is commonly isolated in appendicitis (7.4% to 16.4%).^[5,7–9]^ The reported isolation rate of *K pneumonia* is 4.8% to 13%.^[5,7,8]^

Although *E coli* showed high susceptibility (96%) to commonly used cephalosporins, quinolones showed 83.4% efficacy. *Enterococcus species*, which are resistant to cephalosporins, require penicillin-based antibiotics. In the present study, *Enterococcus species* was frequently observed in the cases of complicated appendicitis, and penicillin-based antibiotics should be considered for complicated appendicitis. *P aeruginosa*, which is not resolved by CMZ, flomoxef, or cefazolin, necessitates broad-spectrum antibiotics such as TAZ/PIPC and MEPN for effective treatment.^[6]^

We also observed that the rate of bacteria culture positivity was higher in the acute appendicitis patients with appendicoliths. Hattori et al reported that the numbers of aerobes and anaerobes were increased in patients with appendicoliths,^[6,9]^ and we thus speculate that the increased number of bacterial species may have been induced by the appendiceal lumen obstruction. In large random controlled trials, the presence of an appendicolith has been shown to be associated with a more complicated course of the disease.^[10]^

In the management of patients with acute appendicitis, the selection of suitable antibiotics plays a significant role, particularly in patients experiencing infectious complications postsurgery and those undergoing nonsurgical treatment. The proper antibiotic choice should encompass agents that demonstrate effectiveness against both facultative and aerobic Gram-negative organisms as well as anaerobic organisms.^[11]^ The commonly employed third-generation cephalosporin for empirical antibiotic treatment has proven efficacy against a wide range of microorganisms, including *E coli.* Our present findings suggest that cephalosporins are generally suitable, with the exception of efficacy against *P aeruginosa*. In cases involving *P aeruginosa*, treatment options such as TAZ/PIPC and carbapenem may be considered.

Globally accepted guidelines such as the 2017 SIS-IDSA guidelines,^[3]^ which are based on the results of clinical trials of antibiotics for intra-abdominal infections including appendicitis, recommend using cefotaxime or ceftriaxone plus metronidazole or ertapenem as the preferred agents for the initial empiric therapy of lower-risk patients. Guidelines and recent reports do not recommend broad-spectrum regimens with activity against *P aeruginosa* and fluoroquinolone-resistant and ESBL-producing bacteria for patients with mild-to-moderate community-acquired infections unless antimicrobial resistance risk factors exist, such as recent antimicrobial exposure, a past infection with a resistant strain, or a high prevalence of resistance in the patient’s community or in recent areas of travel.^[3,12]^ However, the 2020 WSES Jerusalem guidelines^[4]^ recommend a single preoperative dose of broad-spectrum antibiotics in patients with acute appendicitis undergoing an appendectomy.

ESBL prevalence in the West Pacific and Southeast Asia (46% and 22%, respectively) exceeds that in Europe and the Americas (4% and 2%, respectively).^[13]^ Furthermore, the incidence of fluoroquinolone-resistant *E coli* in Asia is notably higher, ranging from 5.7% to 32.8%, compared to Europe (1.4–23.3%) and North America (2.8–8.9%).^[14]^ In Japan, ESBL-producing *E coli* and fluoroquinolone-resistant *E coli* constitute 9.5% to 15% and 15.6% to 30% of all *E coli* isolates, respectively.^[7,15]^ In this study, the frequency of ESBL-producing *E coli* was 8.8%, and the frequency of fluoroquinolone-resistant *E coli* was 11.1%. While these rates fell below those reported in previous studies, they were not insignificant. Yukumi et al^[7]^ reported third-generation and fourth-generation CEPs or fluoroquinolone-based regimens seem to not be appropriate as a first choice in Japan or other Asian countries. The prevalence of ESBL and fluoroquinolone-resistant *E coli* in Asia challenges the applicability of these guidelines, thereby emphasizing the importance of region-specific considerations.

Our study has certain limitations to consider. It was a retrospective analysis involving a relatively small cohort (n = 141) and was conducted exclusively at a surgical department within a local community hospital in Japan. Consequently, the findings are specific to our regional context and may undergo alterations over time. A comprehensive assessment of different antibiotics should be conducted in a prospective trial.

## 5. Conclusion

Because antibiotic administration has emerged as a reasonable first-line treatment for patients with uncomplicated appendicitis, an understanding of the role of patient’ microbiological profiles and antibiotic susceptibilities in acute appendicitis is of substantial importance. Studies from Asian countries such as Japan can deepen our understanding of regional antibiotic resistance patterns and guide effective therapeutic strategies.

## Author contributions

**Conceptualization:** Hiroe Kitahara.

**Data curation:** Hiroe Kitahara, Yonfan Park, Kai Seharada, Masaki Yoshimura, Yukihiko Karasawa.

**Formal analysis:** Hiroe Kitahara.

**Funding acquisition:** Hiroe Kitahara.

**Investigation:** Hiroe Kitahara.

**Methodology:** Hiroe Kitahara.

**Project administration:** Hiroe Kitahara.

**Resources:** Hiroe Kitahara.

**Writing – original draft:** Hiroe Kitahara.

**Writing – review & editing:** Hiroe Kitahara.

**Visualization:** Akira Horiuchi.

## References

[1] Podda M Cillara N Di Saverio S.; ACOI (Italian Society of Hospital Surgeons) Study Group on Acute Appendicitis. Antibiotics-first strategy for uncomplicated acute appendicitis in adults is associated with increased rates of peritonitis at surgery. A systematic review with meta-analysis of randomized controlled trials comparing appendectomy and non-operative management with antibiotics. Surgeon. 2017;15:303–14. 28284517 10.1016/j.surge.2017.02.001

[2] Bruno Leonardo BL Matthias M Melanie L. The role of intraoperative swab during appendectomy in patients with uncomplicated and complicated appendicitis. J Colorectal Dis. 2023;38:272. 10.1007/s00384-023-04566-8 PMC10665244 37991592

[3] Mazuski JE Tessier JM May AK. The Surgical Infection Society revised guidelines on the management of intra-abdominal infection. Surg Infect (Larchmt). 2017;18:1–76. 28085573 10.1089/sur.2016.261

[4] Di Saverio S Podda M De Simone B. Diagnosis and treatment of acute appendicitis: 2020 update of the WSES Jerusalem guidelines. World J Emerg Surg. 2020;15:27. 32295644 10.1186/s13017-020-00306-3 PMC7386163

[5] Dae WS Byung KP Suk WS. Bacterial culture and antibiotic susceptibility in patients with acute appendicitis. Int J Colorectal Dis. 2018;33:441–7. 29488087 10.1007/s00384-018-2992-z

[6] Hattori T Yuasa N Ikegami S. Culture-based bacterial evaluation of the appendix lumen in patients with and without acute appendicitis. J Infect Chemother. 2019;25:708–13. 30982727 10.1016/j.jiac.2019.03.021

[7] Yukumi S Ishimaru K Suzuki H. Appropriate antibiotic selection during the in-hospital waiting period for surgery for appendicitis. J Anus Rectum Colon. 2022;6:259–63. 36348947 10.23922/jarc.2022-016 PMC9613419

[8] Son JT Lee GC Kim HO. Routine intraoperative bacterial culture may be needed in complicated appendicitis. Ann Coloproctol. 2020;36:155–62. 32674546 10.3393/ac.2019.11.04.1 PMC7392572

[9] Garzon-González LN Padilla LT Patiño F. Association between bacterial resistance profile and the development of intra-abdominal abscesses in pediatric patients with perforated appendicitis: Cohort study. Pediatr Surg Int. 2023;40:18. 38082019 10.1007/s00383-023-05570-3 PMC10713695

[10] Flum DR Davidson GH Monsell SE.; CODA Collaborative. A randomized trial comparing antibiotics with appendectomy for appendicitis. N Engl J Med. 2020;383:1907–19. 33017106 10.1056/NEJMoa2014320

[11] Jeon HG Ju HU Kim GY. Bacteriology and changes in antibiotic susceptibility in adults with community-acquired perforated appendicitis. PLoS One. 2014;9:e111144. 25343342 10.1371/journal.pone.0111144 PMC4208803

[12] Talan DA Saltzman DJ DeUgarte DA. Methods of conservative antibiotic treatment of acute uncomplicated appendicitis: a systematic review. J Trauma Acute Care Surg. 2019;86:722–36. 30516592 10.1097/TA.0000000000002137 PMC6437084

[13] Karanika S Karantanos T Arvanitis M. Fecal colonization with extended-spectrum beta-lactamase-producing enterocbacteriaceae and risk factors among healthy individuals. Clin Infect Dis. 2016;63:310–8. 27143671 10.1093/cid/ciw283

[14] Stapleton AE Wagenlehner FME Mulgirigama A. *Escherichia coli* resistance to fluoroquinolones in community-acquired uncomplicated urinary tract infection in women: a systematic review. Antimicrob Agents Chemother. 2020;64:e00862–20. 32747356 10.1128/AAC.00862-20 PMC7508571

[15] Wada K Yokoyama T Uno S. Nationwide surveillance of bacterial pathogens isolated from patients with acute uncomplicated cystitis in 2018: conducted by the Japanese Research Group for Urinary Tract Infections (JRGU). J Infect Chemother. 2021;27:1169–80. 33863634 10.1016/j.jiac.2021.03.012

